# Phospholipid-Based Topical Nano-Hydrogel of Mangiferin: Enhanced Topical Delivery and Improved Dermatokinetics

**DOI:** 10.3390/gels9030178

**Published:** 2023-02-24

**Authors:** Faisal K. Alkholifi, Aftab Alam, Ahmed I. Foudah, Hasan S. Yusufoglu

**Affiliations:** 1 Department of Pharmacology, College of Pharmacy, Prince Sattam Bin Abdulaziz University, Al Kharj 11942, Saudi Arabia; 2 Department of Pharmacognosy, College of Pharmacy, Prince Sattam Bin Abdulaziz University, Al Kharj 11942, Saudi Arabia; 3 Department of Pharmacognosy & Pharmaceutical Chemistry, College of Dentistry & Pharmacy, Buraydah Private Colleges, Buraydah 51418, Saudi Arabia

**Keywords:** dermal bioavailability, dermal pharmacokinetics, topical administration, nanoemulsion, microemulsion, anticancer, breast cancer

## Abstract

Mangiferin is a herbal drug that has proven anticancer potential. Owing to its lower aqueous solubility and poor oral bioavailability, the full pharmacological potential of this bioactive drug has not fully been explored. In the present study, phospholipid-based microemulsion systems were developed to bypass oral delivery. The globule size of the developed nanocarriers was less than 150 nm and the drug entrapment was >75% with a drug loading ~25%. The developed system offered a controlled release pattern following the Fickian drug release. This enhanced mangiferin’s in vitro anticancer activity by four-fold, the cellular uptake was observed to be improved by three-fold on the MCF-7 cells. Ex vivo dermatokinetic studies showed substantial topical bioavailability with a prolonged residence time. The findings provide a simple technique to administer mangiferin via a topical route promising a safer, topically bioavailable and effective treatment option for breast cancer. Such scalable carriers with immense topical delivery potential may provide a better option for present-day topical products of a conventional nature.

## 1. Introduction

Mangiferin, chemically known as 2-β-D-glucopyranosyl-1,3,6,7-tetrahydroxy-9H-xanthen-9-one, is extracted from the seed, peel and kernel of *Mangifera indica* and other plants of higher order. This plant possesses immense potential for managing breast cancer, as reported in the literature. Recent scientific studies have established the beneficial effects of mangiferin, not only limited to immunomodulation, lipid-lowering, anticancer, antioxidant, antidiabetic, antiallergic and antimicrobial effects, but have been further reported for many lifestyle-related disorders [1,2,3]. The anticancer potential of this bioactive molecule has been explored by researchers all over the globe for benefits in breast, colon, lung and neuronal cancers. This phytoconstituent acts by various mechanisms ranging from free radical scavenging to the induction of apoptosis [4]. It is an acceptable trend that phytochemicals are explored for multiple therapeutic benefits [5,6], but numerous challenges are associated with the delivery of phytochemicals [7,8].

Like most phytochemicals, mangiferin is associated with problems such as higher lipophilicity, lesser solubility in biological fluids and poor bioavailability [9]. The oral bioavailability of mangiferin is reported to be lower than 2% [10]. Numerous reports are available where emulsifying systems have enhanced the bioavailability of poorly bioavailable drugs [11]. Drug delivery carriers such as adhesive nanocarriers [12], PEGylated carbon nanotubes [10], injectable hydrogels [13], nano-mixed micelles [14] and nanostructured lipid carriers [15] have been developed by researchers to deliver this promising drug by one route or another.

Since oral delivery represents a challenge, it was envisioned that topical formulations were developed employing the principles of drug delivery and biocompatible components such as phospholipids [16]. Phospholipids, made of biocompatible material and an integral part of biological membranes, were selected as an important component for developing microemulsion-based gel [17]. Microemulsion systems are known to properly dissolve and deliver the drug across the skin barriers [18]. Therefore, it was envisioned to explore the effect of phospholipid-incorporated microemulsion-based topical gel on the topical bioavailability of this promising phytochemical and provide a proof-of-concept to the scientific fraternity along with the possible biological evaluations. However, researchers have utilised microemulsions for topical delivery [18,19], but for mangiferin, no such topical product has been explored for the anticancer potential. A few attempts have been made to develop the nanoemulsions of this bioactive and explore its potential in inflammation and skin regeneration. The materials, process and approach of the previously published literature are different from the present research, vouching for the novelty of the current work [20].

## 2. Results and Discussion

### 2.1. Construction of Pseudo-Ternary Phase Diagrams

**Figure 1:**
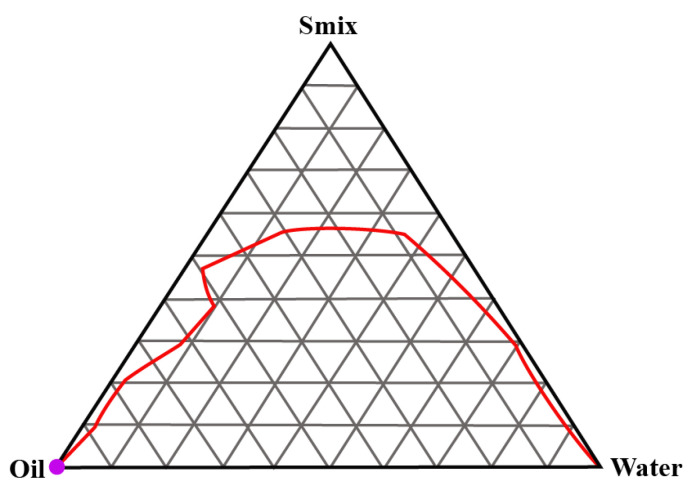
Pseudoternary phase diagram between IPP, water and S_mix_ (1:1). The red line shows the boundary between the monophasic and biphasic region. The area above redline represents the monophasic region.

**Figure 2:**
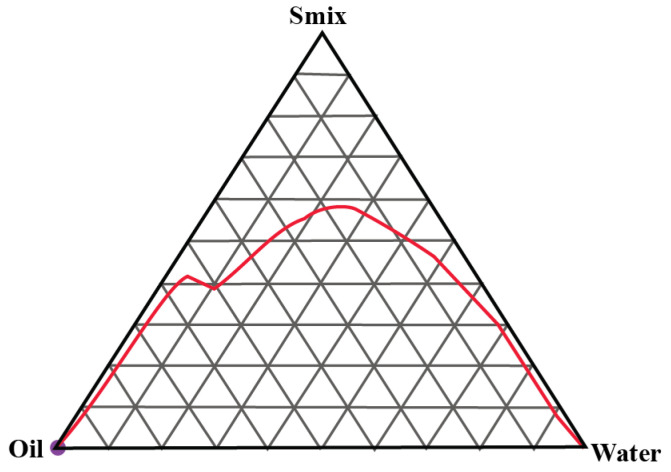
Pseudoternary phase diagram between IPP, water and S_mix_ (2:1). The red line shows the boundary between the monophasic and biphasic region. The area above redline represents the monophasic region.

**Figure 3:**
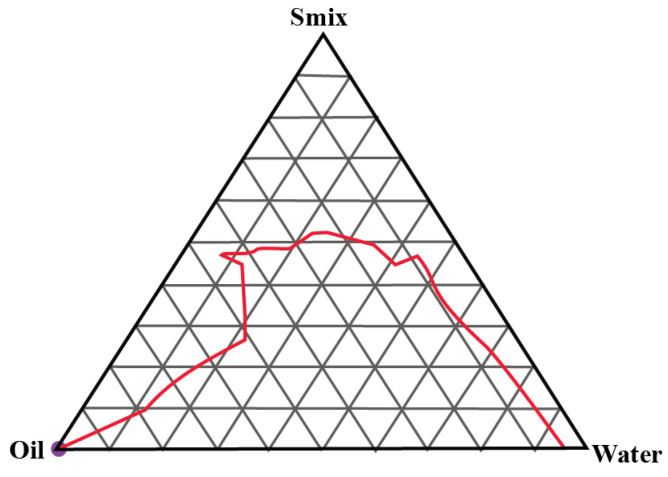
Pseudoternary phase diagram between IPP, water and S_mix_ (3:1). The red line shows the boundary between the monophasic and biphasic region. The area above redline represents the monophasic region.

A total of three pseudo-ternary phase diagrams were constructed. Isopropyl palmitate (IPP) was used as the oil and Gelucire 44/14 and Labrasol were used as the surfactant and cosurfactants in S_mix_ ratios of 1:1, 2:1 and 3:1, respectively. Figure 1, Figure 2 and Figure 3 show the pseudoternary phase diagrams prepared with IPP, water and S_mix_ ratios of 1:1, 2:1 and 3:1, respectively.

The monophasic area obtained in all three ternary phase diagrams was relatively wider in range. It invariably increased with the increase in the surfactant-to-cosurfactant ratio. Labrasol, a polyethylene glycol derivative of capric acid and caprylic acid triglycerides, offers excellent emulsification properties for various oils. Gelucire 44/14 is composed of polyoxylglycerides with well-established emulsification attributes [21,22]. From these pseudoternary phase diagrams, a total of 9 formulations were selected and preceded further.

### 2.2. Optimisation of the Microemulsion Composition

The nine selected formulations were characterised for particle size, drug entrapment (% EE) and drug loading (% DL). The details of the obtained results are presented in Table 1.

The globule size of the selected formulations was below 300 nm and invariably for every oil composition; it was least for the 2:1 surfactant-to-cosurfactant ratio with the least value for the formulation F8. The 2:1 Smix ratio offered better emulsification to all the oil compositions resulting in a smaller globule size. The total formulation-to-drug ratio was of approximately 6.7. Therefore, the entrapment efficiency for each formulation was >75% with the maximum drug entrapment offered by formulation F3. A pattern analogous to the globule size was also observed in drug entrapment. For each oil composition, the drug entrapment was lower at both the 1:1 and 3:1 Smix ratios and the highest at the intermediate ratio of 2:1. For drug loading, a similar pattern was also observed, which was the best for F8 followed by F2. Based on the least globule size and highest entrapment efficiency/drug loading, formulation F8 was selected as the optimised formulation.

### 2.3. Polydispersity, pH, Zeta-Potential and Morphology

**Figure 4:**
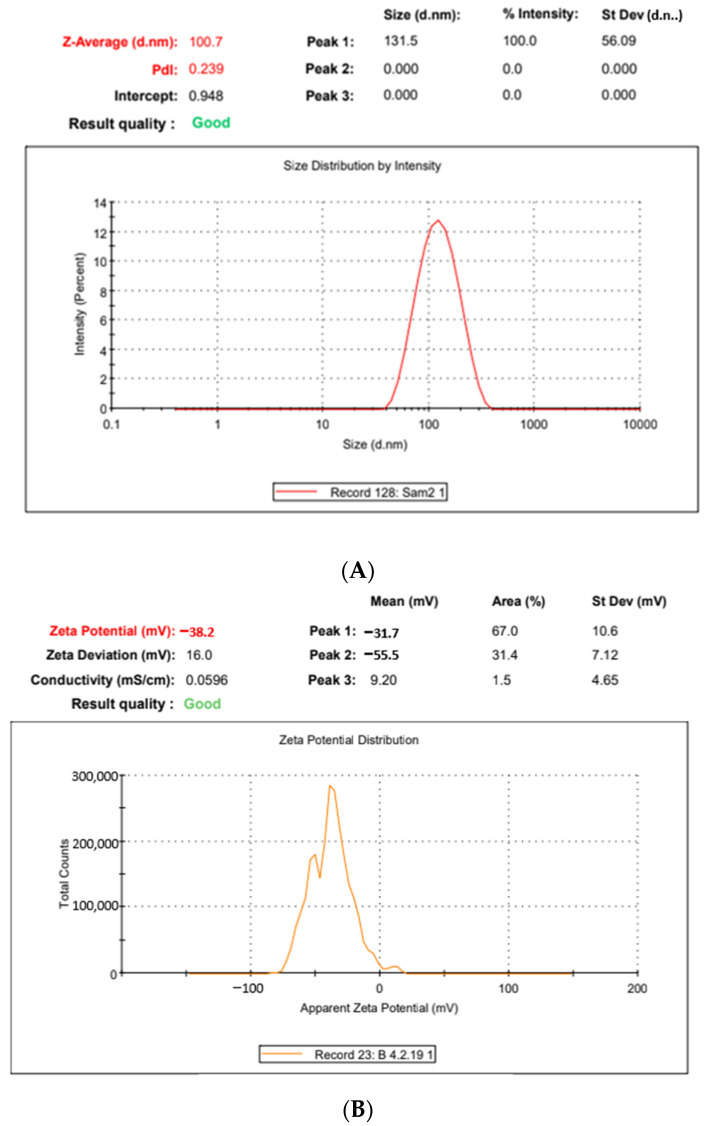
(**A**): The globule size, polydispersity index and globule size distribution of the formulation F8. (**B**): The zeta-potential graph of the formulation F8.

The globule size of the optimised microemulsion formulation was 100.7 ± 18.7 nm with a PDI value of 0.239. The lower size range of globules assured a nanoemulsion formulation with a PDI confirming the reliability of the micromeritic data of the dispersed phase. The zeta potential of the developed nanodispersion derived from the F8 formulation was −38.2 ± 7.46 mV, assuring substantial dispersion stability [23,24]. The particle size and zeta potential results are shown in Figure 4A,B, respectively.

**Figure 5:**
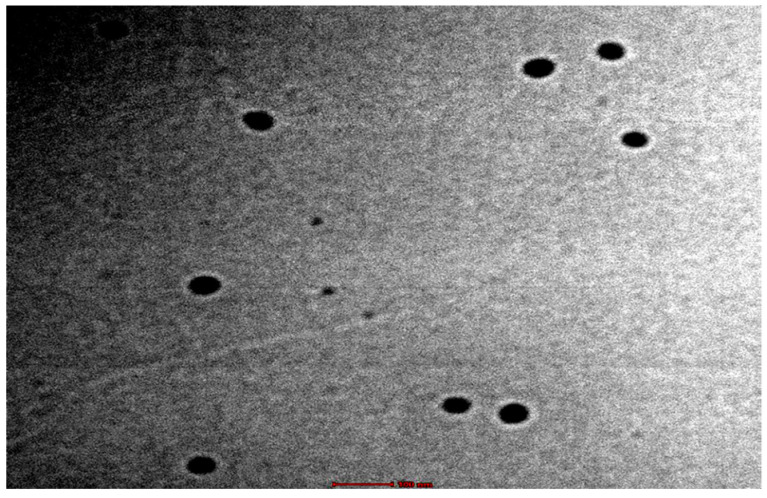
The TEM microphotograph of the microemulsion formulation F8.

The transmission electron microscopy confirmed the formation of spherical globules devoid of any aggregation, as shown in Figure 5. The pH of the selected formulation was 6.92 ± 0.21, which is within the range for the topical products that are well tolerated on the skin [25].

### 2.4. Fourier Transform Infrared Spectroscopy

**Figure 6:**
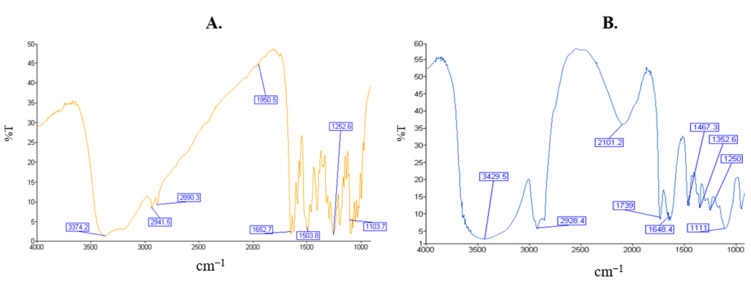

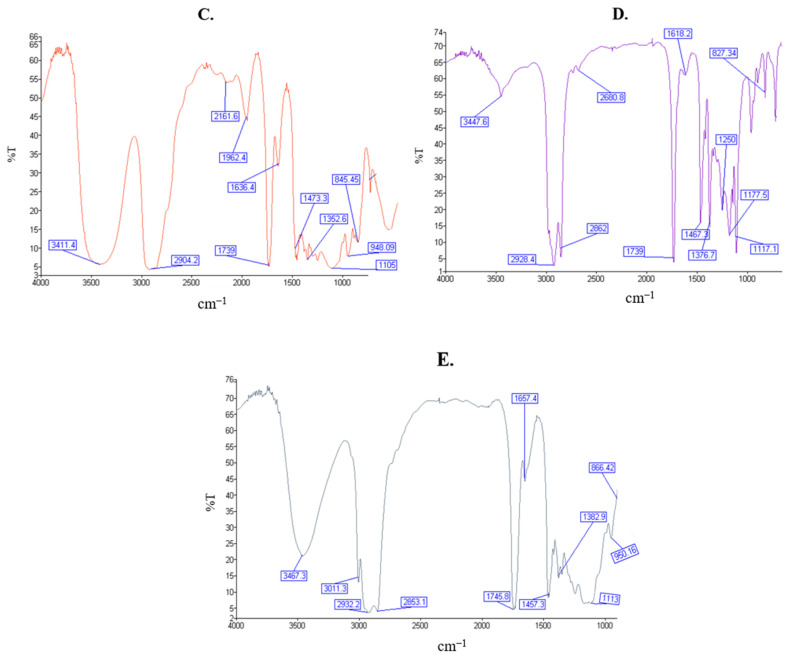
FT-IR spectrum of: mangiferin (**A**), microemulsion (**B**), Labrasol (**C**), IPP (**D**) and Gelucire (**E**).

The results of the FT-IR are shown in Figure 6. Figure 6A represents the FT-IR spectrum of mangiferin with secondary hydroxyl peak at 3374 cm^−1^; anti-symmetric C–H stretching at 2941; symmetric C–H stretching at 2890 cm^−1^; C=O stretching at 1652 cm^−1^; CH–CH bending at 1502 cm^−1^; and C–C stretching at 1103 cm^−1^. The microemulsion (Figure 6B) peaks appeared for the OH- bond at 3429 cm^−1^, C–H stretch at 2928 cm^−1^; C=O stretch at 1739 cm^−1^; C=O bending at 1648 cm^−1^; C–H bending at 1467 cm^−1^; and C-O stretching at 1111 cm^−1^, indicating no interactions between the drug and the excipients, or even just among the excipients. Figure 6C represents the FT-IR spectra of Labrasol with the characteristic C–H stretch 2904 cm^−1^; C=O stretch 1739 cm^−1^; C–H bending at 1473 cm^−1^; and C–O stretch 1103 cm^−1^. Figure 6D shows the FT-IR spectrum of the oil with the O–H stretch at 3447 cm^−1^; C–H stretching asymmetric 2928 cm^−1^; and C–H symmetric 2862 cm^−1^; C=O stretch at 1739 cm^−1^; and C–O stretching at 1117 cm^−1^. Figure 6E represents the FT-IR spectra of the Gelucire showing characteristic C–H stretch 2932 cm^−1^, C=O stretch 1745 cm^−1^ and C–O stretch 1113 cm^−1^.

### 2.5. Incorporation in the Hydrogel

The final composition of the nano-hydrogel was 0.02 g of mangiferin/g of the gel, 0.1578 g of oil/g of gel, 0.6316 g of S_mix_, 0.0125 g of Carbopol, 934/g of gel, 0.0125 g of triethanolamine/g of gel, 0.01 g of phospholipid/g of gel and 0.1556 g of water/g of gel. The conventional hydrogel contained 0.02 g of mangiferin/g of gel, 0.0125 g of Carbopol 934/g of gel, 0.0125 g of triethanolamine/g of gel, 0.0125 g ethanol/g of gel and 0.9425 g of water/g of gel.

### 2.6. Rheological Studies of the Nano-Hydrogel

**Figure 7:**
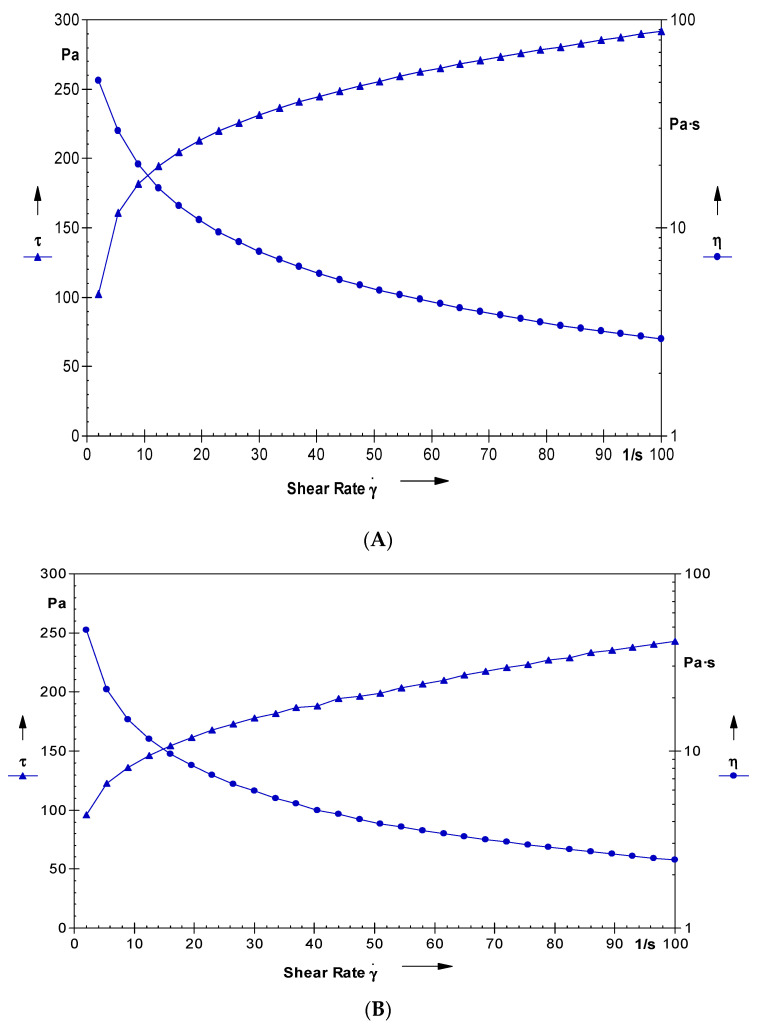
(**A**): The graph showing the variation in viscosity and shear stress with the varying values of shear rate for the developed nano-hydrogel. (**B**): The graph showing the variation in viscosity and shear stress with the varying values of shear rate for the developed conventional gel.

The graph between the shear stress and shear rate is shown in Figure 7A. The graph shows the variation in the shear stress as a function of shear rate. It is clear that, with an increase in the shear rate, the shear stress of the system increased. The system offered a non-Newtonian behaviour with a plastic flow and offered a yield value of 50 Pa. The average viscosity of the developed system was 8.7 Pa·s. The studies clearly vouch that the developed system behaved as a Newtonian after the yield value. Such behaviour is desired for the gelled system and is suitable for storage in tubes [26]. The rheological profile of the conventional hydrogel is provided in Figure 7B. The viscosity (8.97 Pa·s) and the yield values (61 Pa) of the conventional gel are higher than that of the developed nano-hydrogel owing to the presence of oil and surfactants in the latter.

### 2.7. Drug Permeation and Drug Retention Studies

**Figure 8:**
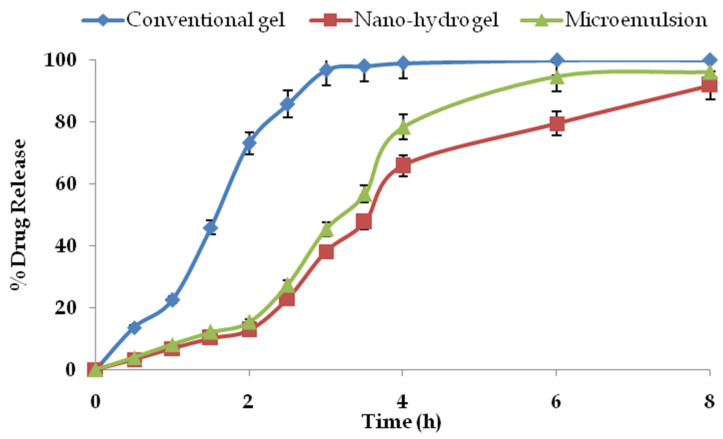
Cumulative drug permeation profiles of mangiferin from the studied systems (n = 3).

The drug permeation profile of mangiferin from the optimised microemulsion, nano-hydrogel formulation and the conventional gel for 8 h is shown in Figure 8. From the obtained data, it is clear that the drug permeation from the conventional gel was without any resistance across the skin. In contrast, the drug release was sustained for the nano-hydrogel formulation. However, the release from the globules of the microemulsion was intermediate. At every time point, there was a significant difference in the release profile from all the systems (*p* < 0.05). The plain drug from the conventional gel was almost completely permeated in 3 h of the study, whereas the nano-hydrogel maintained the drug release for 8 h, advocating a sustained release pattern.

On the other hand, the microemulsion could also appreciably control the drug release vis-à-vis the conventional gel. However, the microemulsion’s permeation rate was higher than the nano-hydrogel. The average drug permeation rate for the conventional hydrogel was 0.323 mg/h, which was significantly higher than the drug encapsulated in the nano-hydrogel with a permeation rate of 0.116 mg/h (*p* < 0.05). The drug permeation rate for the microemulsion was 0.139 mg/h, which was significantly higher than the nano-hydrogel and lower than the conventional formulation (*p* < 0.05). The gelling of the nanocarrier resulted in a more controlled release pattern from the microemulsion. The average permeation rate confirmed the controlled release pattern from the nano-hydrogel and drug-loaded microemulsion, whereas the drug permeation mechanism from the plain drug gel was of first order.

**Figure 9:**
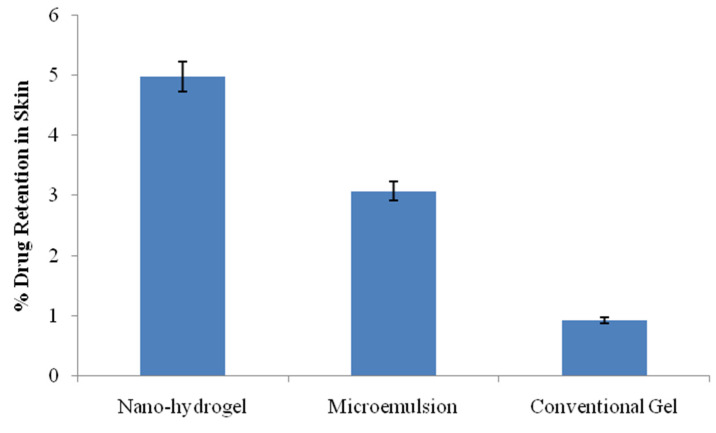
Drug retention in the skin from the studied formulations (n = 3).

The drug retention in the skin is shown in Figure 9. The drug retained in the skin for the developed nano-hydrogel was 4.98 ± 0.02%, whereas for the conventional gel, the drug retained in the skin was 0.92 ± 0.01%, whereas the drug retention in the skin from the ungelled microemulsion was 3.07 ± 0.02%. The drug retention in skin layers by the nano-hydrogel was substantially higher than the drug retention by plain gel as well as the ungelled microemulsion, although the values for the microemulsion were significantly higher than the conventional gel (*p* < 0.05). The plausible reasons are the composition and nano-architecture of the microemulsion which resulted in the better adhesion, fusion and depot formation in the skin, which was further facilitated by gelling. Such deposition is desired in topical delivery as the drug will be released from the depot and assured drug concentrations for longer duration [18,27].

### 2.8. Cancer Cell Viability

**Figure 10:**
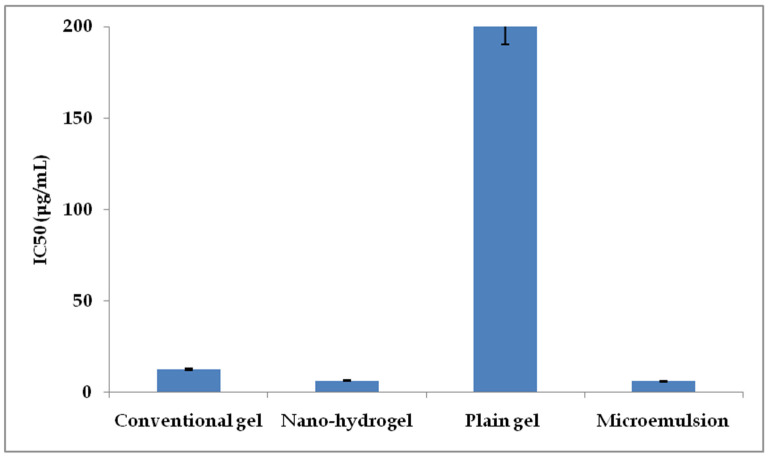
IC_50_ values of the tested formulations against MCF-7 cell lines.

MTT-based cell cytotoxicity assay was performed on MCF-7 cells. The IC50 values for the plain mangiferin gel (conventional gel) and the nano-hydrogel-incorporated mangiferin were obtained to be 12.5 μg/mL and 6.25 μg/mL, respectively. On the other hand, the plain gel formulation was found to exhibit no significant toxicity, even at the concentrations above 200 μg/mL. Interestingly, the IC50 value of the ungelled microemulsion was found to be of the lowest magnitude, i.e., 5.98 μg/mL. The results are shown in Figure 10. The IC50 values obtained for the drug were in consonance with the previously published results [28]. The substantial decrease in the IC50 value of mangiferin after the encapsulation in microemulsion gel exhibited 100% enhancement in the anticancer activity due to the easy penetration and better availability of drugs as a result of components such as IPP, Gelucire and Labrasol [19,22]. However, the ungelled microemulsion had better cytotoxicity than the nano-hydrogel in the in vitro cytotoxicity assays. This was because the ungelled microemulsion was a low-viscosity liquid that could easily get inside the cancer cells; consequently, the cells were more exposed to the microemulsion.

### 2.9. Dermatokinetic Studies

**Figure 11:**
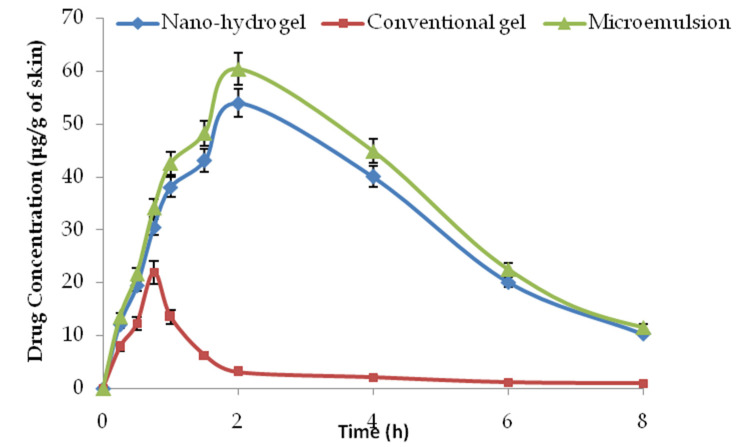
Mean plasma mangiferin concentration versus time graph in rodents (n = 4).

The results obtained from the dermal pharmacokinetic studies are shown in Figure 11 and Table 2. As shown in the figure, the drug concentration offered by the nano-hydrogel formulation in skin was substantially higher than the drug concentration from the plain drug gel at every time point (*p* < 0.05). As a poorly absorbed drug, the substantially higher drug concentrations in rodents skin from the nano-hydrogel is a significant achievement [29]. The drug-loaded microemulsion was not the final product for topical application, and the ungelled microemulsion was also studied for comparison. The performance of the microemulsion was slightly better than its gelled version, owing to the apparent reason for diffusion limitation in the gelled system. However, for better retention, the in vivo gels are better. There was 2.5-fold enhancement in the Cmax and an approximately eight-fold improvement in the area under the curve (AUC). The studies clearly demonstrated the enhanced dermal bioavailability potential of a promising drug using biocompatible components such as IPP, Gelucire and Labrasol. The microemulsion-based nano-hydrogel not only improved the topical bioavailability of the drug, but also improved the half-life and bioresidence of the drug molecule in the dermal/epidermal compartments.

## 3. Conclusions

The developed microemulsion-based nano-hydrogel formulation with sub-micron size and acceptable zeta-potential not only improved the anticancer activity of mangiferin by two-fold, but also improved the topical bioavailability of this promising bioactive many times. Though the drug was not yet explored in the developed system for topical delivery, the present study provides an option to deliver the drug by topical route with inherent promises such as enhanced permeation, retention, conducive dermal pharmacokinetic profile and improved anticancer potential. Such scalable products using simple preparatory techniques with substantial beneficial outcomes for safety, efficacy and dermal pharmacokinetics provide a ray of hope for the further exploration of such products for better outcomes. A significant understanding of the underlying principles of dermatokinetics, as in this case, can result in the better location of the drug. It can assist in achieving the targets of targeted delivery.

## 4. Materials and Methods

### 4.1. Materials

Mangiferin and 3-(4,5-dimethylthiazol-2-yl)-2,5-diphenyltetrazolium bromide (MTT) were purchased from Sigma-Aldrich, St. Louis, MO, USA. The hydrochloric acid, chloroform, isopropyl palmitate (IPP) and methanol was obtained from SDFCL Chem. Limited, India, whereas the buffer reagents, i.e., disodium hydrogen phosphate, sodium chloride and potassium dihydrogen phosphate were supplied by CDH Pvt. Ltd., New Delhi, India. Gelucire 44/14, Labrasol ALF and Labrafil M 1944 CS were procured from Gattefossè, Lyon, France. The acetonitrile was obtained from Spectrochem, Mumbai, India. Phospholipid (Phospholipon 90 G) was procured from Lipoid, Ludwigshafen, Germany.

### 4.2. Methods

#### 4.2.1. Construction of Pseudo-Ternary Phase Diagrams

Series of pseudo-ternary phase diagrams were obtained by the titration method, in which a mixture of oil (isopropyl palmitate) and Smix (surfactant and cosurfactant, i.e., ratios of Gellucire 44/14 and Labrasol) were titrated with water and vice versa. For Smix, three mass ratios of Gellucire 44/14 and Labrasol were prepared in 1:1, 2:1 and 3:1. Various mixtures of water and Smix in the ratios of 1:9, 2:8, 3:7, 4:6, 5:5, 6:4, 7:3, 8:2 and 9:1 were prepared and titrated with oil. For instance, water and S_mix_ were mixed in the weight ratios ranging from 9:1 to 1:9, and every ratio was titrated with oil. During titration, a small volume of oil was added to the mixture and vortexed. Titration was continued until the visual observation of haziness. The volume was noted and all the volumes were converted into mass percentage. Every titration point was plotted on the ternary phase diagram depicting the boundary between the homogeneous and heterogeneous phases. Analogously, various ratios of oil and Smix were prepared and titrated with water until it appeared hazy. The phase diagrams was prepared with the obtained mass percentage data [18,30].

#### 4.2.2. Optimisation of the Microemulsion Composition

From the three developed pseudo-ternary phase diagrams, a total of 9 formulations were prepared, based on the pseudo-ternary phase diagrams. These formulations were coded from F1 to F11. From all the ternary phase diagrams, three oil concentrations were selected, i.e., 12.5%, 15.78% and 22.22%. The composition of the systems is shown in Table 3.

The amount of mangiferin was kept constant at the concentration of 2% *w*/*w* in consonance with the previously published reports [31]. These 9 formulations were characterised for various attributes that have been discussed in the subsequent sections. Based on the globule size, various microemulsion parameters and the maximum entrapment efficiency, one formulation was selected for further studies.

#### 4.2.3. Determination of Drug Entrapment Efficiency and Drug Loading

To determine the entrapment efficiency of the developed microemulsion, the dialysis method was employed. In brief, each formulation equivalent to 1 mg of drug was packed in a dialysis bag and sealed. The dialysis bag was suspended in 30.0 mL of methanol and kept for stirring at 50 rpm for 2 h. The dialysis fluid was analysed for the cumulative unentrapped drug diffused from the developed system at the end of the study. The drug entrapment efficiency was reported as the amount of entrapped drug per hundred parts of the theoretical drug. On the other hand, the drug loading was reported as the amount of drug encapsulated per hundred parts of the drug carrier [10,24]. The formulae for the drug entrapment and drug loading were as follows:

$$Drug\;entrapment\;efficiency=\frac{\left(Total\;drug-Diffused\;Drug\right)}{Total\;Drug\;}\times 100$$

$$Percent\;drug\;loading=\frac{Entrapped\;drug}{Total\;carrier\;to\;entrap\;the\;drug}\times 100$$

#### 4.2.4. Micromeritics, pH, Zeta-Potential and Morphology

The developed formulations were subjected to particle size, particle size distribution and zeta-potential determination using Zetasizer (Nano ZS 90, Malvern, UK). The measurements were recorded in triplicate and the average value was reported as the result. For morphology, transmission electron microscopy was employed using H7000 model (Hitachi Tokyo, Japan). The samples were stained with 1% phosphotungstic acid and placed over carbon-coated copper grid. Systronics pH meter was employed to determine the pH of the undiluted formulations. The pH meter was calibrated with standard buffered solutions over the pH range of 4.0–7.0 and the recordings were made in triplicate, without any dilution.

#### 4.2.5. Fourier Transform Infrared Spectroscopy

The Fourier transform infrared spectroscopy (FT-IR) of various samples were performed on Spectrum 3 FT-IR Spectrometer (PerkinElmer, Waltham, MA, USA). In brief, the samples were punched in potassium bromide pellets and scanned over the wavelength range of 200 cm^−1^ to 4000 cm^−1^. Liquid samples were adsorbed on the potassium bromide tablets. Interpretations were made using standard reference materials and published reports [32].

#### 4.2.6. Incorporation of the Nanosystem in Hydrogel

Phospholipid (equivalent to 1% *w*/*w* of the final formulation) was dispersed in water using a magnetic stirrer at 100 rpm to obtain a milky dispersion. A stock of 10% *w*/*w* Carbopol 934 was prepared in water-dispersed phospholipid and stored overnight. An equal amount of triethanolamine was added drop-wise and the gel was neutralised. The requisite amount of hydrated gel was added in the selected microemulsion, and properly mixed to obtain the desired gel with a Carbopol concentration of 1.25% *w*/*w* [26]. For the preparation of the conventional gel, mangiferin (2% *w*/*w*) was dispersed in ethanol (1.25% *w*/*w*) and Carbopol gel was incorporated by mixing. The swollen gel was neutralised with triethanolamine (1.25% *w*/*w*) and the final Carbopol content was 1.25 % *w*/*w*.

#### 4.2.7. Rheology of the Nano-Hydrogel

The developed nano-hydrogel was characterised for the rheological attributes using a Paar Physica cub and bob rheometer at 37 °C. On average, 5 g of the developed gel was placed in the cup of the rheometer and allowed to equilibrate. The shear stress studied range was 0.1–100 per second and was automatically increased by the software of the equipment, after dipping the bob into the cup. The recordings of the respective shear stress at a particular shear rate were employed for the construction and interpretation of a rheogram. From the rheogram, parameters such as the average viscosity and yield value were determined [33].

#### 4.2.8. Ex Vivo Drug Permeation and Drug Retention Studies

For the ex vivo skin permeation studies and drug retention studies, the excised skins of healthy Laca mice were employed. The methodology and execution of the skin permeation studies and dermatokinetics on rodent skin were duly approved by the Standing Committee of Bioethics Research, Prince Sattam bin Abdulaziz University Al-Kharj, Saudi Arabia, (SCBR-024-2022). To excise the skin, the rodents were sacrificed by cervical dislocation and the skin was harvested. After the removal of the hair using depilatory cream, the skin was washed thrice with normal saline. The hairless skin was mounted over the donor compartment of the Franz diffusion cell and placed in such a way that its inner side touched the diffusion medium of the receptor compartment. The diffusion medium employed was 30 mL of phosphate-buffered saline of pH 6.8 containing 1% of Tween 80. Plain mangiferin in Carbopol gel (conventional gel), microemulsion and the developed nano-hydrogel were applied on the upper side of the mouse skin in triplicate and the samples from the donor compartment were withdrawn at predetermined time-points. To maintain the sink volume, an equal volume of diffusion medium was replenished after each sample. After filtration, the samples were analysed by HPLC as reported by Allaw et al. [34]. The drug permeation was determined by dividing the amount of drug permeated by the total amount and multiplying it by 100. After the completion of the skin permeation studies, the skin from the donor compartments was removed and washed thrice with water to remove any traces of adhered formulation. The skin was excised into small pieces and placed in measured amount of ethanol overnight for the complete extraction of the drug. After filtration, the amount of drug retained in the skin was determined using HPLC [34,35]. The developed system offered a controlled release pattern following the Fickian drug release and governed by the following equation:

$$j=D\frac{\left(C_{d}^{0}-C_{a}\right)}{X}$$

In the above equation, *j* is the flux of the drug, $C_{d}^{0}$ is the freely dissolved unentrapped drug concentration outside the developed system in the donor compartment, *C_a_* is the acceptor drug concentration, *X* is the thickness constant and *D* is the diffusion coefficient.

#### 4.2.9. Cancer Cell Viability Assay and Normal Cell Safety

The in vitro anticancer activity of the developed system and the plain drug was evaluated on MCF-7 breast cancer cell lines. The cells were cultured in 96-welled plates for 48 h with a supply of 5% carbon dioxide. The cells were treated with various concentrations of the formulations and the conventional gel and incubated for 24 h. To each well, 20 µL of MTT (5 mg/mL) was instilled and incubated for 4 h. In each well, 200 μL of dimethyl sulfoxide was added to dissolve the formazan crystals. The absorbance values were recorded at 560 nm [10].

#### 4.2.10. Dermal Pharmacokinetic Studies

The dermatokinetic studies were performed analogous to the skin permeation studies, except that, for each time point, one Franz cell was employed. The method reported by Raza et al. was slightly modified [36,37]. The dermis and epidermis were not separated in the present study, but the whole skin was used. It was extracted in ethanol and the drug contents were analysed for that very time point. Analogously, the skin for every time point was processed. The drug amounts for each time point were subjected to one compartment open-body model and various vital dermatokinetic parameters such as first-order permeation rate constant (Kp), the first-order elimination rate constant (Ke), area under the curve (AUC), maximum achievable concentration (Cmax) and the time required to reach Cmax (Tmax) were determined.

## Figures and Tables

**Table 1:** The characterisation of various developed microemulsion formulations.

Code	F1	F2	F3	F4	F5	F6	F7	F8	F9
Size (nm)	286.59	254.35	278.20	185.73	179.34	210.62	118.55	100.7	114.91
% EE	78.29	82.33	82.17	76.87	81.75	79.94	76.09	83.43	82.18
% DL	11.74	12.41	12.32	11.53	12.26	11.99	11.41	12.51	12.32

**Table 2:** The dermal pharmacokinetic parameters obtained from the skin drug concentration–time profile of mangiferin in rodent skin.

Dermatokinetic Parameter	Conventional Gel	Microemulsion	Nano-Hydrogel
C_max_ (μg/g of skin)	21.97 ± 1.98	60.45 ± 4.11	53.98 ± 5.07
T_max_ (h)	0.75	2	2
${\mathrm{AUC}}_{0}^{\infty }$ (μg·mL^−1^·h)	41.55 ± 9.03	361.31 ± 36.13	328.46 ± 23.18
Kp (h^−1^)	1.91	5.01	4.67
K(h^−1^)	0.43	0.28	0.29
T_1/2_ (h^−1^)	1.61	2.48	2.38

**Table 3:** Composition of 9 formulation compositions for optimisation.

Formulation Code	F1	F2	F3	F4	F5	F6	F7	F8	F9
Oil (%)	12.5	15.78	22.28	12.5	15.78	22.28	12.5	15.78	22.28
Ternary Phase (S_mix_)	1:1	1:1	1:1	2:1	2:1	2:1	3:1	3:1	3:1
S_mix_ (%)	43.75	42.10	38.88	58.33	56.15	51.85	65.62	63.16	58.33
Water (%)	q.s. to 100	q.s. to 100	q.s. to 100	q.s. to 100	q.s. to 100	q.s. to 100	q.s. to 100	q.s. to 100	q.s. to 100

## Data Availability

Not applicable.
